# Suspension Culture of *Polyscias filicifolia* L.H. Bailey Cells as a Superproducer of Very Long Chain Saturated Fatty Acids, or an Unusual Phenomenon of Plant Cell Lipid Metabolism

**DOI:** 10.1002/cbdv.71925

**Published:** 2026-11-14

**Authors:** David A. Yankovsky, Artem A. Fomenkov, Maria V. Titova, Roman A. Sidorov

**Affiliations:** ^1^ K.A. Timiryazev Institute of Plant Physiology Russian Academy of Sciences Moscow Russia; ^2^ Faculty of Biology Lomonosov Moscow State University Moscow Russia

**Keywords:** fatty acids, lipids, plant cell cultures, *Polyscias* *filicifolia*, very long chain fatty acids

## Abstract

Plant suspension cell cultures are predominantly exploited for the production of secondary metabolites, whereas their fatty acid (FA) composition remains largely unexplored. In this study, we discovered that a suspension cell culture of *Polyscias filicifolia* L.H. Bailey acts as a hyperproducer of very‐long‐chain fatty acids (VLCFAs) (up to 40% of FA of total lipids (TL) and 35 mg/g dry weight (DW) — a phenomenon that has not been previously observed *in vivo* in plants or in plant cell cultures. Using gas chromatography–mass spectrometry (GC‐MS) analysis of dimethyloxazoline derivatives, we identified 22 monoenoic, 2 dienoic, 1 trienoic, and one previously undescribed polyunsaturated FA containing an acetylenic bond. We confirmed that VLCFAs are not covalently bound in suberin but are constituents of glycerolipids. Over the cultivation cycle, the major VLCFA (22:0) remained stable until the degradation stage, where it increased sharply, while classical unsaturated FA declined. The strong negative correlation between VLCFAs and classical FAs was observed, indicating distinct physiological roles. The *P. filicifolia* suspension culture represents a promising biotechnological source of VLCFAs for industrial applications.

## Graphical abstract

The *Polyscias filicifolia* cell culture demonstrates an anomalously high level of very‐long‐chain fatty acids (VLCFAs) unrelated to suberin synthesis. A strong negative correlation between VLCFA and classical FAs accumulation is established, alongside the identification of a novel acetylenic acid. This work presents an innovative plant source for commercial lipid production.

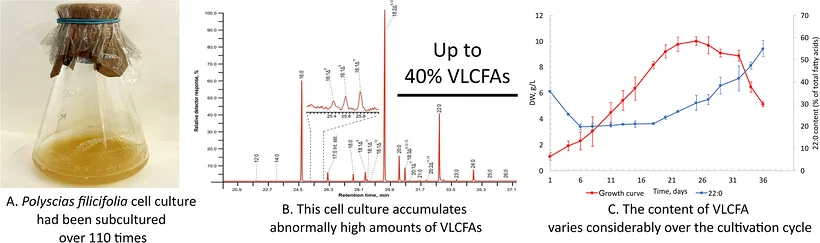

**Abbreviations**
BHT: butylated hydroxytoluene
DMOX: dimethyloxazoline derivatives of fatty acids
DW: dry weight
ECL: equivalent chain length
FA: fatty acid
FAME: fatty acid methyl ester
GC‐MS: gas chromatography–mass spectrometry
TL: total lipids
TLC: thin‐layer chromatography
TMSH: trimethylsulfonium hydroxide
VLCFA: very‐long‐chain fatty acid

## 1 Introduction

Plant cell cultures have a wide application in producing various beneficial secondary metabolites, including the antitumor drug paclitaxel and the antiarrhythmic drug ajmaline [1]. The cultivation of suspension plant cell cultures in bioreactors renders commercial use a possibility. Following the extraction of substances of interest, a significant quantity of unused biomass remains, containing lipids, which are ordinarily not extractable under conditions of isolation of polar secondary metabolites and proteins. A significant number of reviews have been dedicated to the importance of lipids for human nutrition and health. Furthermore, the composition of lipids in the diet has been shown to be a significant factor [2, 3, 4]. Consequently, the analysis of the lipid composition of plant cell cultures is essential for the acquisition of lipids that possess beneficial nutraceutical, cosmetic, and pharmacological properties.

The lipid metabolism of plant cell cultures is not yet fully elucidated. It has been established that the fatty acid (FA) composition of total lipids (TL) is analogous to that of the intact plant. The main FAs of TL of cultures are palmitic (16:0), stearic (18:0), oleic (18:1Δ^9^), linoleic (18:2Δ^9,12^), and α‐linolenic (18:3Δ^9,12,15^) acids [5, 6]. However, in addition to the main FAs present in cultures, there are also unusual ones, such as petroselinic [7] and malvalic [8] acids. It is evident that all of these examples of FAs are limited to a chain consisting of 18 carbon atoms, although there are FAs with a longer chain length.

Very long chain fatty acids (VLCFAs) are a class of FAs characterized by having more than 20 carbon atoms and serving as precursors for numerous metabolites biosynthesis [9]. Plant cells could contain a variety of saturated and unsaturated monoenoic, dienoic, and even polyenoic FAs with a very long chain [10]. They are found both in neutral and polar lipids, but are more used for wax and suberin synthesis, so are essential for protection against biotic and abiotic types of stress [11, 12]. It is a general rule that VLCFA does not accumulate in cell cultures in quantities exceeding 5% [13, 14].

VLCFA are of industrial importance. For instance, in 2017, Xu et al. developed a method for producing diesel‐like biofuel from docosanoic acid [15]. In this regard, the identification of cell cultures capable of VLCFA super production could have potential commercial value.

During the analysis of the FA composition of TLs of suspension cell cultures from the All‐Russian Collection of Cultured Plant Cells [16], we discovered that a cell culture of *Polyscias filicifolia* L.H. Bailey (*Araliaceae*) was able to accumulate levels of VLCFAs in TLs that were significantly above the range typical for *P. filicifolia* leaves or other cell cultures [5, 11, 13]. As this phenomenon has not yet been described in the scientific literature, we decided to study in detail the FA composition and FA metabolism during the cultivation of *P*. *filicifolia* cells. This was done in order to discover whether VLCFA biosynthesis is associated with the stages of culture growth, as well as to identify the stages when this synthesis is most intense.

## 2 Results and Discussion

### 2.1 Fatty Acid Composition of Total Lipids of Leaves and Suspension Cell Culture

To determine whether the accumulation of VLCFAs is a species‐specific trait of *P. filicifolia*, the FA composition of TL from the cell culture and leaves of this plant was studied. The results of the FA composition analysis of these samples are presented in Table 1.

**TABLE 1:** The analysis of the fatty acid composition of the suspension culture of cells and leaves of *P. filicifolia*. The standard deviation (SD) did not exceed 0.5% for major peaks and 3%–5% for minor peaks. The fatty acid composition and content of total lipids were determined by GC‐MS using margaric acid as an internal standard.

FA	Leaves	Cell culture^a^
FA of Total LIPIDS, %		
16:0	16.3	21.3
18:0	4.9	1.0
18:1 Δ^9^	4.7	1.1
18:2 Δ^9,12^	43.3	36.0
18:3 Δ^9,12,15^	24.0	3.0
20:0	0.5	6.5
22:0	0.3	25.5
24:0	0.6	3.7
Minors^[^ ^b^ ^]^	5.3	1.8
Unsaturation index^c^		
	1.653	0.822

^a^ FAs were determined in the stationary phase on the 22nd day of cultivation.

^b^ 12:0, 14:0, 16:1Δ^7^, 16:1Δ^9^, 18:1Δ^11^, 21:0, 23:0, 25:0 were also recorded, with a content of less than 1%.

^c^ The unsaturation index of total lipids was calculated using the following formula: UI = (1 × (% monoenoic) + 2 × (% dienoic) + 3 × (% trienoic)) / 100.

The high unsaturation index of autotrophic leaf tissues, equal to 1.653, is due to the presence of significant quantities of 18:3Δ^9,12,15^ (24.0%) in this sample, which is associated with its accumulation in thylakoid membrane glycolipids [17]. The suspension cell culture exhibited significantly lower unsaturation indices (0.822). This finding is explained by the presence of considerable quantities of VLCFAs in this specimen, as a result of which the proportion of unsaturated FAs is diminished. The total VLCFAs content amounted to 39.9% in the cell culture, whereas only 1.5% was detected in the leaves.

### 2.2 Accumulation of VLCFAs is not Related to the Synthesis of Suberin

The high VLCFAs accumulation in the cell culture could be explained by the presence of suberin [18]. Since suberin is susceptible to hydrolysis upon heating with aqueous‐alcoholic alkali, a condition that was employed to obtain free fatty acids (FFAs) in the analysis, this can account for the detection of VLCFAs in the cell culture samples [19]. Furthermore, suspension cell cultures have been reported to possess the capacity for suberin synthesis [20].

To confirm or refute the hypothesis that VLCFAs accumulate in suspension culture cells as monomers of suberin, TLs of the *P. filicifolia* culture were extracted using the Bligh–Dyer method and subsequently used for the preparation of FA methyl esters (FAMEs). It is important to note that suberin cannot be extracted by this method [21]. FAMEs were obtained by transesterification using two methods: with trimethylsulfonium hydroxide (TMSH) and with 1% (v/v) sulfuric acid in methanol. In both cases, heptadecanoic acid (17:0) was added to the samples as an internal standard prior to methylation. The chromatograms obtained from the experiment are presented in Figure S1. In both cases, a peak corresponding to the methyl ester of VLCFA was present on the chromatograms, allowing us to reject the assumption that its residues were part of suberin. Like methanol, TMSH is capable of transesterifying lipids; however, unlike methanol, it does not methylate free FA, as evidenced by the absence of a peak corresponding to the methyl ester of 17:0. Thus, VLCFAs in the samples were not in free form but were constituents of cell lipids.

### 2.3 Profile of Major and Minor Fatty Acids of Total Lipids

After confirming the presence of VLCFAs in the lipids, we decided to determine the profile of major and minor FA, as well as to characterize their chromatographic parameters. For this purpose, the corresponding 4,4‐dimethyloxazoline (DMOX) derivatives were prepared from the FFAs, allowing reliable determination of the double bond positions based on their mass spectra [22]. The results of GC‐MS analysis of the DMOX derivatives of FAs from the *P. filicifolia* culture, along with the calculated equivalent chain length (ECL) values and retention times of the corresponding methyl esters, are presented in Table S1. The FA of TL profile of the *P. filicifolia* culture is shown in Figure 1.

**FIGURE 1:**
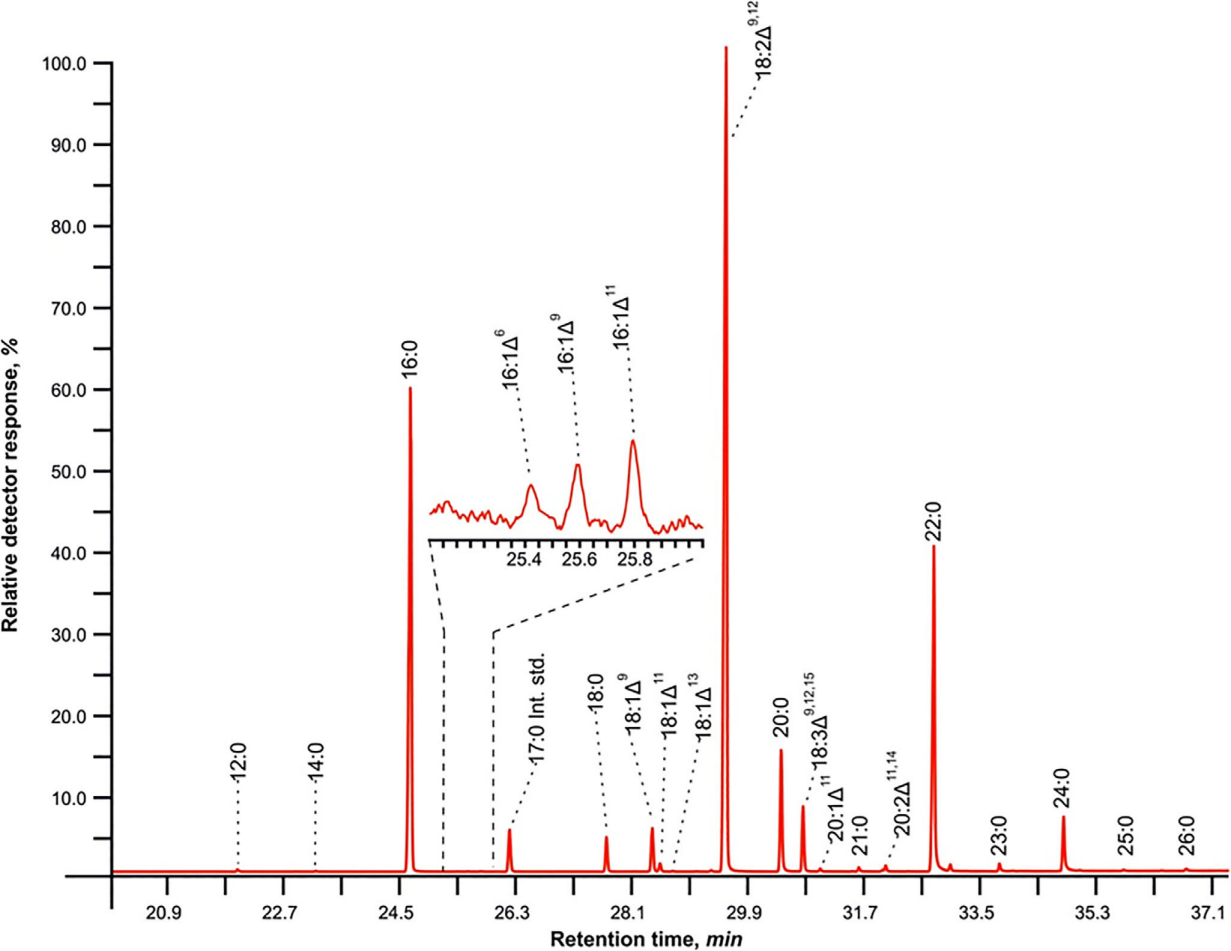
GC‐MS chromatogram of the separation of fatty acid methyl esters derived from total lipids of *P. filicifolia* suspension culture cells (total ion current). The inset shows a magnified view of the chromatogram from 25.4 to 25.8 min. Heptadecanoic acid (17:0) was used as an internal standard (Int. std.).

The analysis revealed 22 monoenoic FAs, 2 dienoic FAs, 1 trienoic FA, and 1 polyunsaturated FA containing an acetylenic bond in a conjugated configuration with an ethylenic bond. Given the absence of data on an acetylenic FA with such a configuration of unsaturated bonds, we believe that it has been identified for the first time. The mass spectrum of the DMOX derivative of 18:3Δ^9^^,^^1^ ^1^ ^a^^,^^1^ ^5^ is presented in Figure 2. The formation of this FA is probably attributable to the metabolism of polyacetylenes, which are derived from FAs and are typical of members of the genus *Polyscias* [23].

**FIGURE 2:**
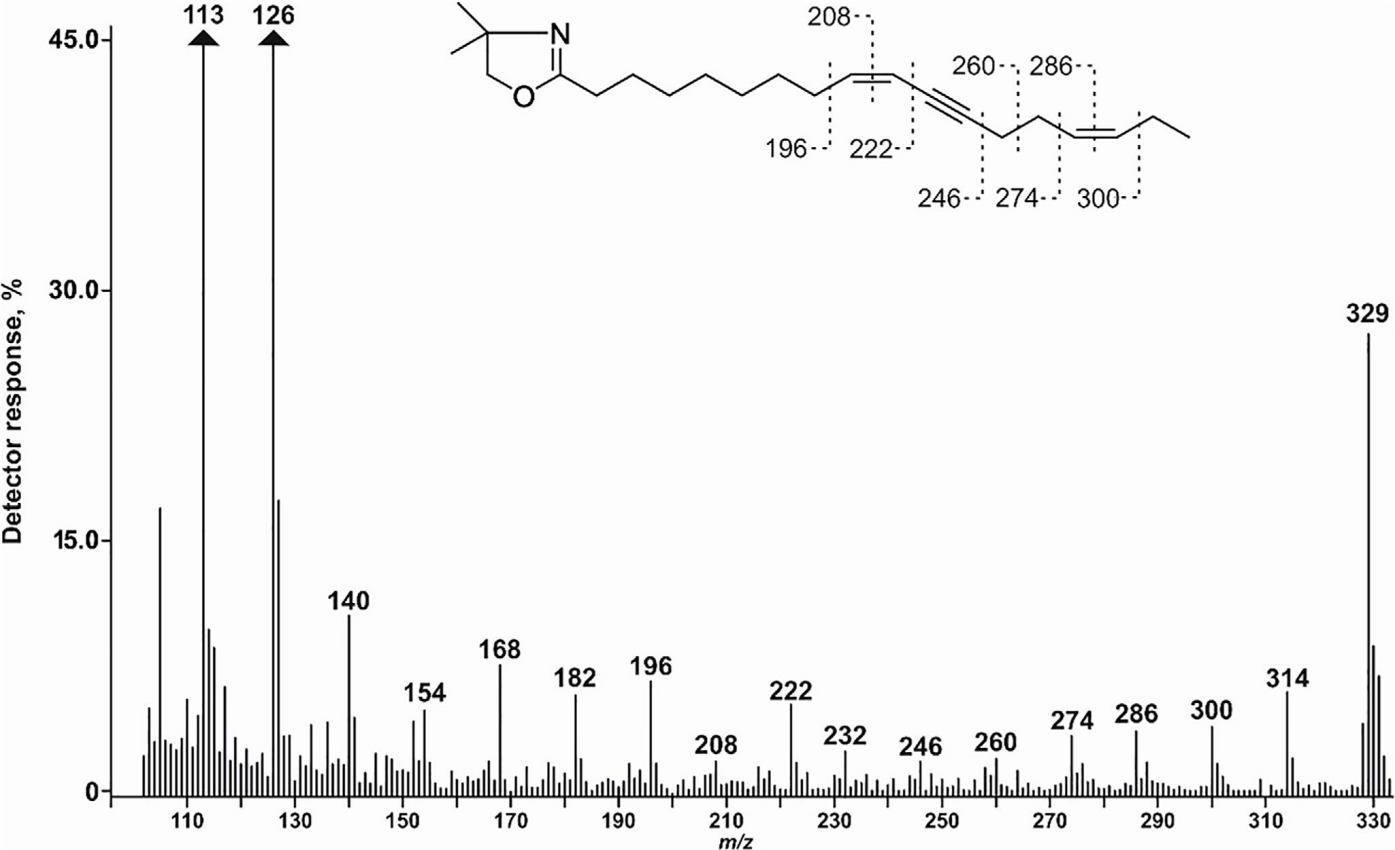
The mass spectrum of DMOX‐derivative of 18:3Δ^9,11a,15^, the newly discovered FA, and the scheme of its fragmentation. The molecular ion [M]^+^ suggests that the DMOX derivative of this FA contains four unsaturated bonds. Mass differences of 12 amu observed for the 208–196 and 286–274 ion pairs indicate the location of double bonds at positions 9 and 15, respectively. A mass difference of 10 amu for the 222‐232 ion pair is consistent with a triple bond at position 11. In contrast, mass differences of 14 amu, typical for methylene (single bond) intervals, are observed for all other ion pairs.

The results of the DMOX derivative analysis allowed us to establish the profile of minor unsaturated FAs and to propose the metabolic relationships between them, involving transformations such as desaturation, elongation, and α‐ or β‐oxidation (Figure 3).

**FIGURE 3:**
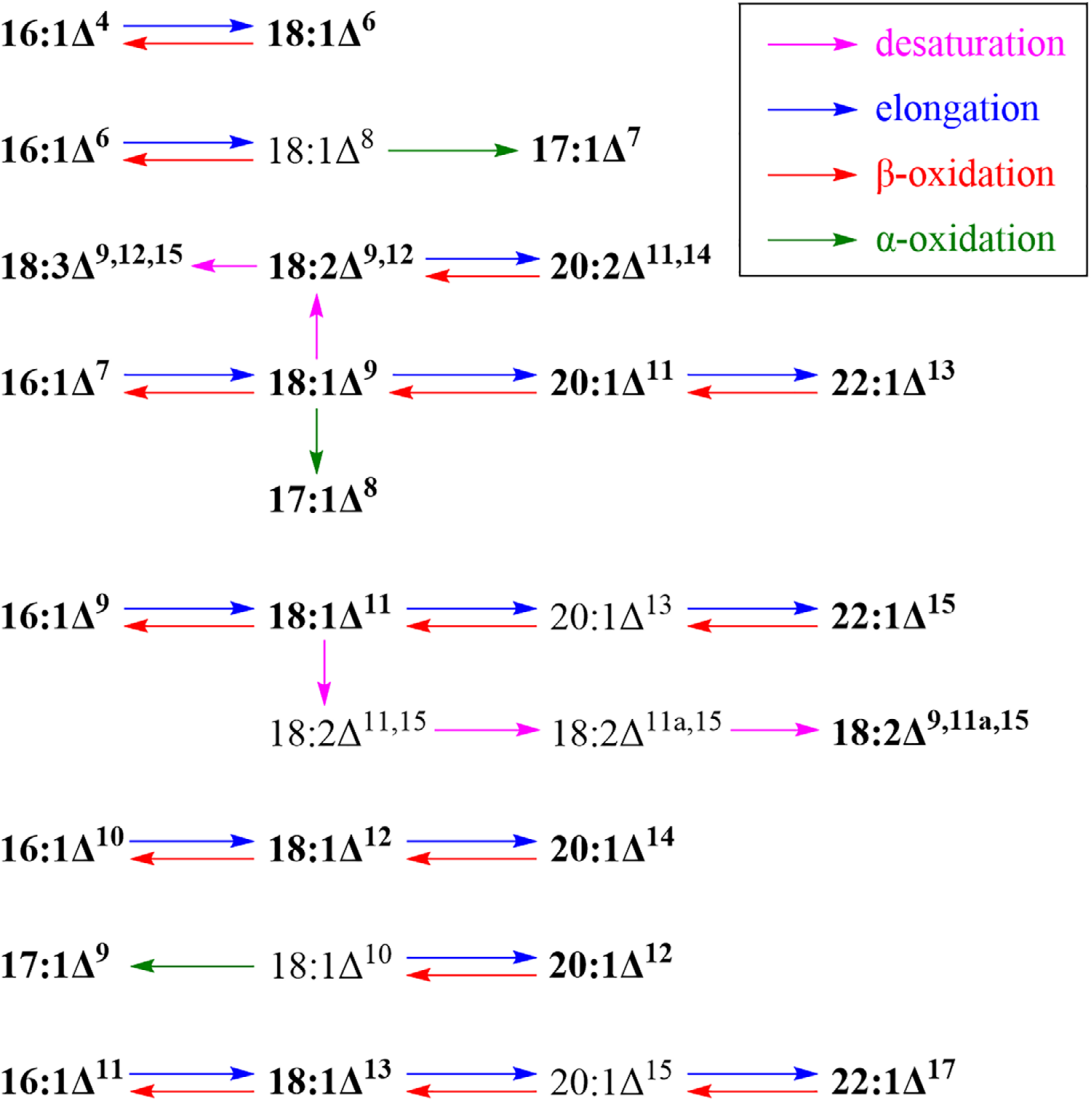
Proposed biosynthetic relationships of unsaturated fatty acids in *P. filicifolia* suspension cell culture. Fatty acids detected by DMOX derivative analysis are shown in bold. In addition to those presented, 16:1Δ^14^ and 22:1Δ^6^ were also identified; however, their biosynthetic homologs were not found.

### 2.4 Changes of Lipid FA Composition in the Cell Culture Over Its Growth Cycle

Following the determination of the TL FA composition of the *P. filicifolia* culture and confirmation of the hypothesis that its cellular lipids are capable of accumulating VLCFAs, the patterns of changes in the total lipid FA composition over the cultivation cycle were investigated. Two independent passages separated by 2 months were analyzed. The results of the measurements are shown in Figure 4. Growth curves for two examined passages were obtained by monitoring changes in cell dry biomass per volume of culture medium over time (g DW/L_medium_). At each sampling time point, the relative proportions of individual FA within the FAs of TL were determined. Additionally, the TL FA content in dry biomass (mg_FA_/g DW) was quantified using the internal standard method.

**FIGURE 4:**
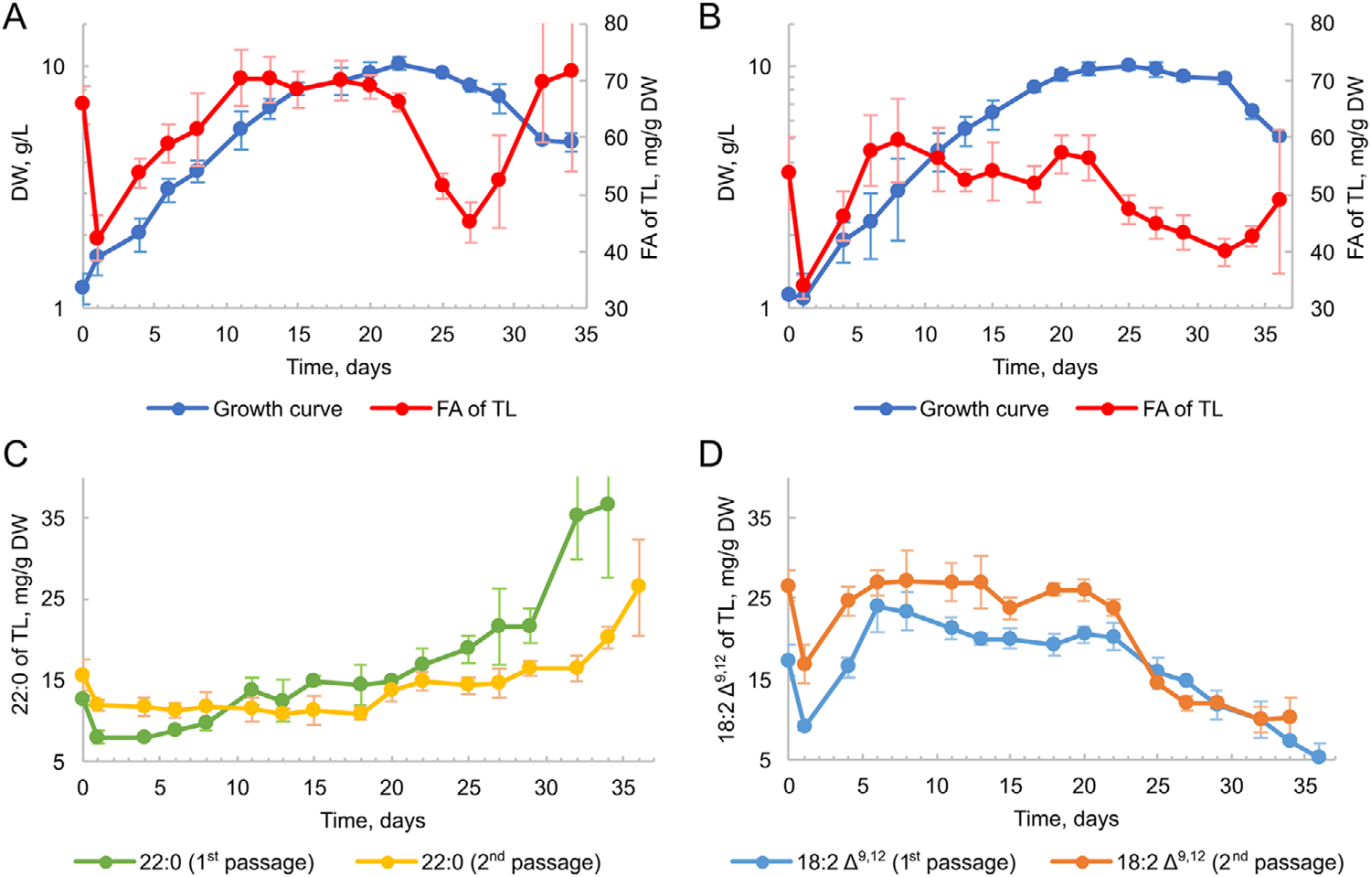
The growth curve and the change in the content of FA of total lipids in the first (A) and second (B) independent passages separated by 2 months. In the lag phase, the content of FA decreased and then increased in the exponential phase. In the stationary phase, the content of FA decreased again, reaching a minimum in the degradation phase. Due to physiological instability in the degradation phase, the values of FA content had large SD. Changes in the content of the main very‐long‐chain fatty acid, behenic acid (C), and the main unsaturated fatty acid, linoleic acid (D), over two passages. Error bars represent the standard deviation (SD).

The lag phase was characterized by a sharp decrease in TL FA content. This decrease is associated, on the one hand, with enhanced metabolic activity and, on the other hand, with an increase in cell volume, which is typical of cells in this phase. During the lag phase, the content of both the major VLCFA (22:0) and the major unsaturated FA (18:2Δ^9^ ^,12^) decreased. However, the content of 22:0 declined more sharply, which may be explained by its role as a storage FA. In contrast, 18:2Δ^9^ ^,12^ remained largely unused due to its involvement in membrane lipids. During the exponential phase, the total fatty acid content increased and reached its maximum in the growth retardation phase. The dynamic of 18:2Δ^9^ ^,12^ was similar. In contrast, the content of 22:0 remained virtually unchanged until the degradation stage. At the degradation stage, a sharp increase was observed in both total FA content and 22:0 content, whereas the content of 18:2Δ^9^ ^,12^ decreased. The increase in total FA content during the exponential phase, reaching a maximum in the growth retardation phase, is likely associated with active biomass accumulation and the formation of storage lipids in preparation for the stationary phase. The lack of significant changes in 22:0 content until the degradation stage suggests that this FA is not involved in ongoing metabolism and may be stored in the form of reserve lipids. The sharp rise in both 22:0 and total FA content observed during the degradation stage may result from the breakdown of nonlipid cellular structures, leading to the release of bound lipids, coupled with a decline in their further metabolic turnover. The decrease in 18:2Δ^9^ ^,12^ content at the same stage is likely attributable to its utilization in stress responses or oxidative degradation.

To test the hypothesis that the physiological role of VLCFAs differs from that of classical cellular FAs, such as 16:0, 18:0, 18:1Δ^9^, 18:2Δ^9^^,^^1^ ^2^, and 18:3Δ^9^^,^^1^ ^2^^,^^1^ ^5^, a Pearson correlation matrix was constructed based on data on changes in the mole fractions of these FA throughout the cultivation cycle (Figure 5A).

**FIGURE 5:**
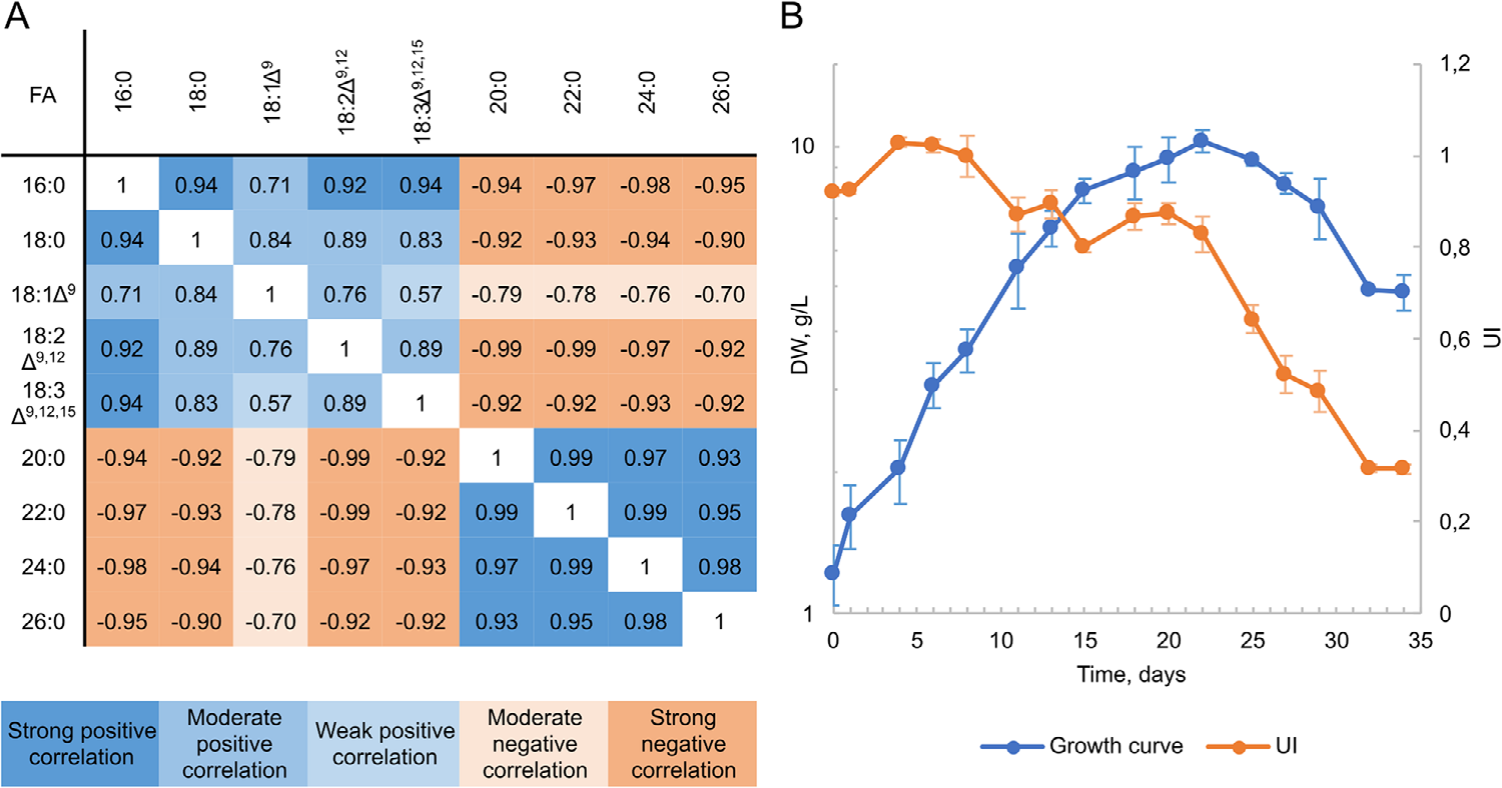
The Pearson correlation matrix based on changes in the FA composition of total lipids (A) and changes in the unsaturation index, UI (B) during the first passage. Error bars represent the standard deviation (SD). VLCFAs are characterized by a strong positive correlation with each other and a moderately strong or strong negative correlation with other FAs. Since the percentage of unsaturated FAs decreases after the stationary phase, and the percentage of VLCFAs increases, there is a significant decrease in the unsaturation index.

The data obtained confirm a strong or very strong negative correlation between the changes in the proportion of VLCFAs and those of other FAs, namely 16:0, 18:0, 18:1Δ^9^, 18:2Δ^9^^,^^1^ ^2^, and 18:3Δ^9^^,^^1^ ^2^^,^^1^ ^5^ — that is, the so‐called “classical” FAs of plant cells. By contrast, VLCFAs show a very strong positive correlation with each other, while the classical FAs demonstrate moderately strong, strong, or very strong positive correlations among themselves. This confirms the difference in the physiological roles of classical FAs and VLCFAs and demonstrates that the dynamics of their proportions are opposite over the cultivation cycle. The rise in VLCFAs content coupled with a decline in unsaturated FAs during the degradation phase results in a marked decrease of the unsaturation index (Figure 5B). Therefore, the accumulation of saturated VLCFAs in cellular membranes appears improbable, as it would lead to an increase in membrane viscosity. These findings point to the likely accumulation of VLCFAs in storage lipids.

## 3 Conclusions

In this study, we demonstrated that the suspension cell culture of *P. filicifolia* accumulates an exceptionally high proportion of VLCFAs, reaching approximately 40% of TL FAs — a level far exceeding the typical 5% reported for most plant cell cultures [24]. We confirmed that VLCFAs are not bound in suberin metabolism but are present as constituents of cellular glycerolipids. These findings support a fundamental difference in the physiological roles of classic cell FAs and VLCFAs. This is evidenced by the difference in the dynamics of changes between VLCFAs and classical FAs over the cultivation cycle. *P. filicifolia* culture thus represents a promising biotechnological source of VLCFAs, including behenic acid, for potential applications in cosmetics and biofuel production.

In further studies, we plan to address two key questions arising from the present work. First, we aim to identify the specific lipid classes in which VLCFAs are localized within *P. filicifolia* cells, as well as the membranes and subcellular compartments in which they reside. Second, we intend to investigate how cultivation conditions (nutrient medium, hormone composition, mechanical stress) influence VLCFA accumulation. Understanding these regulatory factors will not only clarify the metabolic control of VLCFA biosynthesis but also enable the optimization of culture conditions for maximal VLCFA production, which is of potential biotechnological interest.

## 4 Experimental Section

### 4.1 Plant Material and Cultivation Conditions

In this study, the suspension culture of *P. filicifolia* (Ridl.) L.H. Bailey (line No. Pf‐SH) from the All‐Russian Collection of Plant Cell Cultures at the Institute of Plant Physiology of the Russian Academy of Sciences was investigated. This culture was obtained in 2019 from a leaf explant. Fresh leaf samples used for the analysis of the FA composition of TLs were provided by the Greenhouse of the N.V. Tsitsin Main Botanical Garden of the Russian Academy of Sciences.

Suspension cell culture was maintained in 250 mL Erlenmeyer flasks filled with 40 mL of modified Schenk and Hildebrandt (SH) liquid medium [13] with 0.5 mg/L 2,4‐dichlorophenoxyacetic acid (2,4‐D), 0.1 mg/L kinetin, 5 mg/L thiamine hydrochloride, 0.5 mg/L pyridoxine hydrochloride, 5 mg/L nicotinic acid, 100 mg/L *meso*‐inositol, and 30 g/L sucrose. The initial density (inoculum) size was set at 1.10–1.30 gDW L^−1^. The subculture cycle was 21 days for maintenance in the collection and 30‐35 days for determining full growth characteristics. The culture was grown on an orbital shaker (95–100 rpm) at 26°C–27°C and 70%–75% relative air humidity in darkness. By the beginning of the study, the *P. filicifolia* cell culture had been subcultured approximately 110 times.

The alterations in FA composition that occur during the culture growth cycle were the focus of a study that involved two consecutive independent passages separated by 2 months. Within each passage, 100 flasks were inoculated simultaneously and cultured for 35 days. For each analysis point, three flasks were randomly selected from the flask pool. Two cell wet weights were collected from each flask for FA composition analysis. Consequently, for each growth point within a passage, there were six replicates and 72 gas chromatography‐mass spectrometry analyses per passage.

Suspension cell culture samples were collected every 1‐2 days starting from the day of inoculation to monitor growth dynamics. For each time point, 3 replicate flasks were harvested, and the assessment was continued for 35 days. The volume of the suspension cell culture from each flask was measured, and the cultures were vacuum‐filtered using a Büchner funnel and a Bunsen flask to separate cell biomass from the liquid medium. The resulting cell biomass was washed thrice with distilled water, blotted dry and weighed to determine fresh weight (FW). The samples were then air‐dried in a Gemlux GL‐FD‐635S drying oven (Gemlux, Russia) at 40°C for 24 h until achieving a constant weight. The dried samples were weighed to determine cell biomass dry weight. Fresh and dry cell biomass concentrations were calculated per liter (g/L). Growth curves of suspension cell cultures were constructed based on dry weight concentrations. Cell viability was determined using bright‐field microscopy by staining 0.2 mL of cell suspension with 20 µL of 0.1% phenosafranine solution. The number of viable (unstained or less than half stained) cultured units (cell aggregates) was counted, with at least 200 aggregates counted in three biological replicates. Cell viability was expressed as a percentage of living aggregates relative to the total number of aggregates. Cell viability during the cultivation cycle varied within the 88%–95% range during the active growth phase, followed by a sharp dropdown after 28‐30 days during the degradation phase [25].

### 4.2 Biomass Fixation of Cell and Extraction of Total Lipids

To analyze changes in the FA composition of TLs in suspension culture cells over the cultivation period, cells were collected from the flasks by filtration as described in Section 4.1, transferred to 2‐mL glass vials containing 500 µL of ethanol, and derivatized on the same day according to Section 4.4.

To perform TL extraction the suspension culture cells (*circa* 5‐8 g of FW) after filtration were transferred to a 250‐mL conical, flat‐bottomed flask for the purposes of storage and subsequent extraction of TLs. In order to fix the cells, a quantity of 50 mL of 2‐propanol, containing 0.001% butylated hydroxytoluene (BHT), was added to the flask. The flask was then heated at a temperature of +75°C for 15 min. Following a cooling period of 20 min, the flask was stored within a refrigerator set at a temperature of −18°C for a maximum duration of 60 days [26].

In order to obtain a TL extract, the cells in 2‐propanol were transferred to 50 mL Eppendorf tubes and the cells were pelleted by centrifugation for 5 min at 2,300 G. Thereafter, the 2‐propanol was collected in a clean flask. Then 26.25 mL of a chloroform:methanol mixture (1:2, v/v) was added to the pelleted cells, and the tube was agitated for 15 min on a BioSan OS‐20 orbital shaker at 250 rpm. Then, 8.75 mL of chloroform were added, and the tube was shaken for a further 15 min. Then 8.75 mL of water was added, the tube was vortexed for 30 s, and centrifuged for 5 min at 2,300×g. The upper phase was then removed, and the lower chloroform phase was carefully pipetted and combined in a flask with 2‐propanol, which was used for cell fixation. The extraction procedure was repeated twice. The extract was subsequently filtered, after which the solvents were removed using a rotary vacuum evaporator. The dried lipids were then redissolved in a chloroform:methanol mixture (2:1) to obtain a solution with a concentration of 100 mg/mL, which was stored in a glass vial at −18°C [27].

### 4.3 Preparation of Free Fatty Acids

In order to accurately determine the composition and amount of FAs either of TLs in cells of the suspension culture, three weighed portions of fresh cells (*circa* 150‐200 mg FW) were taken from two randomly selected flask from the pool of flasks in the passage. Therefore, a total of six samples were obtained for each growth point within a single passage. In the case of leaves, they were homogenized in a mortar. Fresh biomass was placed into a 2 mL glass vial, and saponification was initiated by adding a solution of 500 µL of 0.001% (m/m) dibutylhydroxytoluene in 96% (v/v) ethanol, along with 120 µL of 5 M aqueous KOH. 100 µL of heptadecanoic acid solution in 2‐propanol (250 µg/mL) was added to the samples as an internal standard. The saponification process was performed for 60 min at a temperature of 75°C using a solid‐state thermostat. Afterwards, to remove unsaponifiable hydrophobic compounds, 600 µL of *n*‐hexane was added to the vial, the mixture was shaken intensively, then centrifuged at 2300 g for 3 min, and the upper phase was removed. The procedure was repeated twice. The free fatty acids (FFAs) of TLs were extracted with two 600 µL portions of *n*‐hexane followed by 150 µL of 20% (m/m) aqueous solution of sulfuric acid. Then, the mixtures were shaken and centrifuged at 2300 g for 3 min. The upper phase containing FFAs was collected in an extraction vial and the procedure was repeated. The upper phases were collected from the two extractions were combined, and the solvent was evaporated to dryness under a stream of argon [24].

### 4.4 Preparation of Fatty Acid Methyl Esters From Free Fatty Acids and Extracted Lipids

To obtain methyl esters of fatty acids (FAME), 200 µL of 1% (*v/v*) sulfuric acid solution in methanol was added to the vial with dried FFAs and methylation was performed for 30 min at 55°С. Then, 100 µL of water and 300 µl of *n*‐hexane were added. The mixture was shaken and centrifuged at 2300×g for 3 min, and the upper phase was collected for GC‐MS analysis. All the procedures described in the Section 4.3,4 were performed in glass chemical utensils on the day of biomass collection. The same method was used for transesterification of FAs from TLs obtained as described in Section 4.2.

In order to obtain reliable evidence that the unusual very long chain FAs were incorporated into glycerolipids rather than in free form, a transesterification reaction was performed on the lipid extract using trimethylsulfonium hydroxide (TMSH), which does not react with free FAs. The experiment was conducted using a freshly prepared 0.2 M TMSH solution in methanol. To this end, a quantity of 4.4 g of trimethylsulfonium iodide was dissolved in 100 mL of methanol, with gentle heating. Subsequently, 5 g of Ag_2_O were added to the flask containing the solution, and the flask was stirred on a magnetic stirrer for a period of 4 h. The solution was then filtered and stored at +4°C for a maximum period of two months [28].

In order to obtain methyl esters from the extract of TLs or individual classes of glycerolipids, 300 µL of a methanolic TMSH solution were added to 1–2 mg of lipid in a 2 mL vial. The vial was then kept for 30 min in a solid‐state thermostat at +55°C [29]. The extraction of methyl esters was conducted using *n*‐hexane, following the addition of 150 µL of water to the mixture, as outlined in Section 4.3.

### 4.5 Concentration of Fatty Acids by Silver TLC

To precisely identify FA isomers of the ethylene bond position, including minor FAs, we preconcentrated fatty acid methyl esters according to their degree of unsaturation by using silver thin‐layer chromatography (TLC), followed by their conversion to DMOX derivatives for GC‐MS analysis.

To this end, silica gel 60 TLC plates were impregnated with a 5% aqueous solution of silver nitrate for 30 min and dried for an hour at 110°C. Then, 5 mg of FAMEs dissolved in *n*‐hexane were applied to the starting line of a 10 × 10 cm plate, and the plate was placed in a chamber with a mobile phase consisting of *n*‐hexane:acetone (90:10 v/v). After the solvent front reached the top of the plate, the plate was air‐dried and sprayed with a solution of 2,7‐dichlorofluorescein in methanol (80 mg/L). Silica gel zones containing FAMEs were visualized under UV light (365 nm). Fractions of mono‐, di‐, and triunsaturated FAs with R_*f*_ values of 0.64, 0.41, and 0.18, respectively, were quantitatively transferred using a spatula into clean vials, and the esters were extracted with 1 mL of a 1:1 v/v mixture of *n*‐hexane:diethyl ether [30].

### 4.6 Preparation of Dimethyloxazoline Fatty Acid Derivatives (DMOX)

The FAMEs obtained as described in Section 4.5. were converted into DMOX derivatives as follows. At first, FAME were saponified to FFAs by KOH using the method described in Section 4.3. The solution of *circa* 1 mg of FFAs was evaporated in an argon flow at 40°С. Then, 100 µL of oxalyl chloride was added. The reaction was carried out for an hour at 50°С. The solution was evaporated and 500 µL of 10% solution of 2‐amino‐2‐methyl‐1‐propanol in methylene chloride was added. The reaction was performed for an hour at 50°С. Then, the mixture was evaporated and 500 µL of trifluoroacetic acid was added. The reaction was also carried out for an hour at 50°С. DMOX derivatives of FFAs were extracted by *n*‐hexane using the method similar to the FAME. Then, they were used for GC‐MS analysis [30].

### 4.7 GS‐MS Analysis of Fatty Acids Derivatives

The capillary GC method was applied for the separation of FA methyl esters. The separation was performed on Crystal 5000.NP gas chromatograph. The analysis was conducted utilizing an AS‐2 M single‐port autosampler, a Chromatec Crystal MSD (Cromatec corp., Russia) mass spectrometric quadrupole detector, and a 60 m Restek Rtx‐2330 capillary column with an internal diameter of 0.25 mm and a stationary phase layer thickness of 0.2 µm. The following conditions were employed: the temperature program was initiated at 60°C for a period of eight minutes, followed by a gradual increase of 10°C per minute until reaching 175°C for a further five minutes. Thereafter, the temperature was increased at a rate of 6,5°C per minute until it reached 245°C, which was maintained for a period of 25 min. The injector temperature was set to 260°C, and the sample was introduced with a split ratio of 20:1. Sample volume was 1 µL. The ion source temperature was set to 230°C, the quadrupole was set to 150°C, and the ionization energy was set to 70 eV. Helium was employed as the carrier gas at a flow rate of 1 mL/min. The mass range scanned was 50–550 𝑚/𝑧. Chromatograms were recorded using the total ion current as the measurement parameter. The peaks of methyl esters were identified by matching their chromatographic parameters (retention time, relative retention time) and mass spectra with the NIST database using AMDIS software [30].

For DMOX derivatives, the initial temperature was set at 140°C for 8 min. It was then increased at a rate of 4°C per min until it reached 210°C, which was maintained for a period of 5 min, the temperature was then increased at a rate of 3°C per min until it reached 250°C for 25 min. Finally, the temperature was increased at a rate of 5°C per min until it reached 240°C for 60 min. All other GC–MS parameters were identical to the FAME preparation method. The mass range scanned was 110–550 𝑚/𝑧.

### 4.8 Statistical Analysis

The normality of all quantitative data was assessed through the application of the Shapiro‐Wilk W‐test. The Pearson correlation criterion was then utilized to ascertain potential relationships between the concentrations of individual FAs during the culture growth cycle. Statistical analysis was performed using Statistica v. 10 software (StatSoft, USA), and data were visualized using SigmaPlot software.

## Author Contributions

**David A. Yankovsky**: collection and fixation of material, sample derivatization, data processing, preparation of the draft article. **Artem A. Fomenkov**: cell fixation, sample collection, discussion and editing of the manuscript. **Maria V. Titova**: cell culturing, supervision of the work, editing the manuscript. **Roman A. Sidorov**: study conceptualization, data curation, GC‐MS analysis.

## Funding

Lipid extraction, GC‐MS analysis, identification of fatty acids, and their quantification were carried out with the support of the Russian Science Foundation (RSF grant no. 21‐74‐30003). Cell cultivation, as well as the collection and fixation of biomass for the study, were performed with the support of the Ministry of Science and Higher Education of the Russian Federation under research project no. 126012615953‐3.

## Conflicts of Interest

The authors declare no conflicts of interest.

## Use of Generative AI and AI‐Assisted Technologies in the Writing Process

The authors did not use AI tools in the preparation of the text and illustrations for this manuscript.

## Supporting information




**Supporting File 1**: cbdv71925‐sup‐0001‐FigureS1.tif

**Supporting File 2**: cbdv71925‐sup‐0002‐TableS1.docx

**Supporting File 3**: cbdv71925‐sup‐0003‐SuppMat.docx

## Data Availability

Raw data and GC‐MS files are available upon request from the corresponding author.
