# Supplementary material for: Suspension Culture of Polyscias filicifolia L.H. Bailey Cells as a Superproducer of Very Long Chain Saturated Fatty Acids, or an Unusual Phenomenon of Plant Cell Lipid Metabolism
Source: Chem Biodivers. 2026 Nov 14;23(11):e71925. doi: 10.1002/cbdv.71925 (PMC13769027; doi:10.1002/cbdv.71925)
Supplement: Supplementary file 1 [file CBDV-23-e71925-s001.docx]

**Supplementary Table 1.** The results of GC-MS analysis of the DMOX derivatives of FAs from the *P. filicifolia* cell culture, retention times (t_R_) of the corresponding methyl esters (ME), and calculated equivalent chain length (ECL) values.

| **FAs** | **M^+^** | **Characteristic ions** | **t_R_ ME** | **ECL**  **ME** |
| --- | --- | --- | --- | --- |
| Monoenoic FAs | | | | |
| 16:1Δ^4^ | 307 | 139, 152, 166 | 24,47 | 16,27 |
| 16:1Δ^6^ | 307 | 126, 167 | 25,12 | 16,33 |
| 16:1Δ^7^ | 307 | 168, 180 | 25,26 | 16,35 |
| 16:1Δ^9^ | 307 | 196, 208 | 25,46 | 16,47 |
| 16:1Δ^10^ | 307 | 210, 222 | 25,58 | 16,63 |
| 16:1Δ^11^ | 307 | 224, 236 | 25,63 | 16,69 |
| 16:1Δ^14^ | 307 | 266, 278 | 25,67 | 16,77 |
| 17:1Δ^7^ | 321 | 168, 180 | 26,43 | 17,3 |
| 17:1Δ^8^ | 321 | 182, 194 | 26,66 | 17,37 |
| 17:1Δ^9^ | 321 | 196, 208 | 26,73 | 17,41 |
| 18:1Δ^6^ | 335 | 126, 167 | 28,05 | 18,13 |
| 18:1Δ^9^ | 335 | 196, 208 | 28,13 | 18,17 |
| 18:1Δ^11^ | 335 | 224, 236 | 28,24 | 18,22 |
| 18:1Δ^12^ | 335 | 238, 250 | 28,33 | 18,25 |
| 18:1Δ^13^ | 335 | 252, 264 | 28,43 | 18,29 |
| 20:1Δ^11^ | 263 | 224, 236 | 30,75 | 20,15 |
| 20:1Δ^12^ | 263 | 238, 250 | 30,87 | 20,27 |
| 20:1Δ^14^ | 263 | 266, 278 | 30,92 | 20,41 |
| 22:1Δ^6^ | 391 | 126, 167 | 32,75 | 22,15 |
| 22:1Δ^13^ | 391 | 252, 264 | 32,97 | 22,28 |
| 22:1Δ^15^ | 391 | 280, 292 | 33,04 | 22,4 |
| 22:1Δ^17^ | 391 | 308, 320 | 33,29 | 22,55 |
|  |  |  |  |  |
| Dienoic FAs | | | | |
| 18:2Δ^9,12^ | 333 | 196, 208, 236, 248 | 29,23 | 19,02 |
| 20:2Δ^11,14^ | 361 | 210, 222, 264, 276 | 31,75 | 20,88 |
| Trienoic FA | | | | |
| 18:3Δ^9,12,15^ | 331 | 196, 208, 236, 248, 276, 288 | 30,49 | 19,85 |
| Acetylenic FA | | | | |
| 18:3Δ^9,11a,15^ | 329 | 196, 208, 222, 246, 274,286 | 34,43 | 22,56 |
