# Supplementary material for: Suspension Culture of Polyscias filicifolia L.H. Bailey Cells as a Superproducer of Very Long Chain Saturated Fatty Acids, or an Unusual Phenomenon of Plant Cell Lipid Metabolism
Source: Chem Biodivers. 2026 Nov 14;23(11):e71925. doi: 10.1002/cbdv.71925 (PMC13769027; doi:10.1002/cbdv.71925)
Supplement: Supplementary file 2 [file CBDV-23-e71925-s003.docx]

**Supplementary Material**

**Supplementary Figure 1**. Total ion current (TIC) GC-MS chromatograms showing the separation of FAMEs derived from total lipids of *P. filicifolia* suspension-cultured cells using two different derivatization methods. The blue trace represents FAMEs prepared using a TMSH solution in methanol, while the red trace represents FAMEs prepared via esterification with 1% sulfuric acid in methanol. Heptadecanoic acid was added to both samples as an internal standard.

**Supplementary Table 1**. Mass spectrometric properties of fatty acid DMOX derivatives identified in the total lipid extract of *P. filicifolia* suspension-cultured cells, alongside the chromatographic properties of their corresponding FAMEs.
